# Psychometric evaluation of a caregiver diary for the assessment of symptoms of respiratory syncytial virus

**DOI:** 10.1186/s41687-018-0036-7

**Published:** 2018-02-21

**Authors:** Valerie Williams, Carla DeMuro, Sandy Lewis, Nicole Williams, Todd Wolynn, Paul Wisman, Stan L. Block, Shelly Senders, Seth Toback, Jason W. Chien

**Affiliations:** 1 RTI Health Solutions, Research Triangle Park, NC USA; 2 Kids Plus Pediatrics, Pittsburgh, PA USA; 3 Pediatric Research of Charlottesville, Charlottesville, VA USA; 4 Kentucky Pediatric & Adult Research, Bardstown, KY USA; 5 Senders Pediatrics, South Euclid, OH USA; 6 United Therapeutics, Research Triangle Park, NC USA; 7 Gilead Sciences Inc, Foster City, CA USA

**Keywords:** Respiratory syncytial virus, Psychometric evaluation, Clinical outcomes assessment, Observer-reported outcome, Caregiver diary

## Abstract

**Background:**

There are no clinical outcome assessment (COA) tools developed in accordance with Food and Drug Administration (FDA) guidance suitable for the evaluation of symptoms associated with respiratory syncytial virus (RSV) infection among infants. The Gilead RSV Caregiver Diary (GRCD) is being developed to fulfill this need; the present research evaluates the GRCD and documents its reliability, validity, and responsiveness among children < 24 months of age with acute RSV infection.

**Methods:**

A prospective, observational study was conducted in the United States during the 2014–2015 northern hemisphere winter season. Subjects were < 24-month, full-term, previously healthy infants with confirmed RSV infection and ≤5 days of symptoms. The GRCD was completed twice daily for 14 days by caregivers. Additional data were collected during the initial visit, subsequent visits, and end-of-study interview. Test-retest reliability (kappa and intraclass correlation coefficients [ICCs]), construct validity (correlations and factor analyses), discriminating ability (analyses of variance and chi-square), and responsiveness (effect sizes and standardized response means) were evaluated.

**Results:**

A total of 103 subjects were enrolled (mean age 7.4 ± 5.3 months). GRCD items were grouped into different subscales according to question content, which, with the exception of the behavior impact domain (ICC = 0.43), demonstrated internal consistency (alphas = 0.78–0.94) and test-retest reliability (ICCs = 0.77–0.94). Hypothesized correlations with parent global ratings of RSV severity ranged from 0.45 to 0.70 and provided support for construct validity. Support for discriminating ability was limited. Effect sizes ranged from − 1.48 to − 4.40, indicating the GRCD was responsive to change.

**Conclusions:**

These psychometric analyses support the validity, reliability, and responsiveness of the GRCD for assessing RSV symptoms in children < 24 months of age.

**Electronic supplementary material:**

The online version of this article (10.1186/s41687-018-0036-7) contains supplementary material, which is available to authorized users.

## Background

Respiratory syncytial virus (RSV) is a common seasonal virus that infects most young children by the age of 2 years and is the leading cause of lower respiratory tract infection requiring hospitalization [1]. More than 80% of all RSV infections are symptomatic with common symptoms among infants, including difficulty breathing, cyanosis, cough, fever, nasal congestion, nasal flaring, rapid breathing, shortness of breath, and wheezing [2]. While mild infections resolve without treatment, more severe cases may require hospitalization and supplemental oxygen, suctioning of mucus from airways, and occasionally intubation with mechanical ventilation [3]. Although no effective treatment exists, there are now several antivirals in development for treatment of RSV infection. A common goal for these medications is to reduce the severity of symptoms and shorten the time to recovery. However, this requires that symptoms of infection be assessed using a reliable tool completed by either a caregiver or health care provider.

While there are tools used to monitor symptoms in RSV-infected pediatric patients, none were developed in accordance with United States (US) Food and Drug Administration (FDA) guidance and therefore are not acceptable for new product labeling. Evidence of content validity, based on patient (or caregiver) input through concept elicitation interviews and cognitive debriefing interviews, is one of the key components of the FDA’s review of clinical outcome assessment (COA) tools. Although the Bronchiolitis Caregiver Diary (BCD) [4] and Canadian Acute Respiratory Illness and Flu Scale (CARIFS) [5] reported qualitative research during the instrument development phase, these two measures targeted populations other than infants and young children < 24 months of age with acute RSV disease. Similarly, psychometric analyses supported the BCD, CARIFS, and Wisconsin Upper Respiratory Symptom Survey (WURSS), but again in different populations [6–8]. Therefore, content validity and psychometric evidence are not available for any of these measures for assessment of RSV symptom severity in children with acute RSV infection and consequently are not considered appropriate for use in clinical trials with the goal of supporting labeling claims for a new therapeutic agent.

In support of the development of antivirals for acute RSV infection in children < 24 months of age, the Gilead RSV Caregiver Diary (GRCD) is an observer-reported outcome (ObsRO) measure designed to assess RSV signs and symptoms from the perspective of caregivers of children (< 24 months of age) with acute RSV [9]. The GRCD items were developed in accordance with the FDA guidance on patient-reported outcomes (PROs), including a review of the literature to evaluate existing COAs and identify constructs of interest, consultation with medical experts, and direct input from caregivers of children in this age group with RSV [9, 10]. In-depth individual interviews with adult caregivers of 16 children < 24 months old with RSV elicited concepts that informed GRCD item development. Following a structured set of item generation principles, candidate daytime and overnight items intended to assess clearly observable signs associated with RSV infection were drafted and subsequently evaluated in iterative rounds of cognitive testing with an additional 23 caregivers (15 nonhospitalized children, 8 hospitalized children) to pretest and refine the draft GRCD questionnaire. These interviews assessed comprehension, refined the wording, optimized the response scales, supported the appropriateness of the recall period, and confirmed the content validity of the items. Therapeutic-area experts provided input throughout the instrument development process [9].

The objectives of this study were to psychometrically evaluate the GRCD and document its reliability and validity in infants and very young children infected with RSV. In addition, the present analyses examined the possibility of item reduction to decrease respondent burden and developed an optimal scoring algorithm for the measure.

## Methods

### Study Design

This was an outpatient, multicenter, prospective, 2-week, US-based observational study of children (< 24 months of age) with a diagnosis of RSV confirmed via rapid antigen diagnostic during a single RSV season, October 2014 to February 2015. No medical procedures or treatments were supplied as part of this study; physicians diagnosed and treated patients per usual practice. Six sites were involved in patient identification and recruitment (Georgia, Kentucky, Ohio, Pennsylvania, Virginia). All subjects were previously healthy, full-term children < 24 months of age seeking their first health care visit for a physician-diagnosed acute respiratory tract infection of ≤5 days of duration. Full study inclusion and exclusion criteria are listed in the Additional file (Additional file 1: Table S1, online).

At visit 1, vital signs, physical exam findings, and demographic information were collected. The RSV clinical severity score was calculated using respiratory rate, oxygen saturation, presence of retractions, and ability to feed [11]. The study physicians assigned each subject a Clinician Global Impression of Severity (CGIS [12]) score of 1 = “normal, not at all ill,” 2 = “borderline ill,” 3 = “mildly ill,” 4 = “moderately ill,” 5 = “markedly ill,” 6 = “severely ill,” or 7 = “among the most extremely ill patients.” Both the Midulla clinical severity score and CGIS were used in the GRCD validity analyses.

For each eligible subject, a single caregiver was instructed to complete the GRCD, Parent Global Impression of Severity (PGIS), and Parent Global Impression of Change (PGIC), the last two of which were used in the validation analyses of the GRCD [13, 14]. The PGIS (“On average, how would you describe your child’s RSV symptoms right now?” 1 = “Mild” 2 = “Moderate,” 3 = “Severe,” or 4 = “Very Severe”) was completed once at the initial visit while the PGIC (“Since the start of the study, my child’s RSV symptoms are ___” 1 = “Very much improved” 2 = “Much improved,” 3 = “Minimally improved,” 4 = “No change” 5 = “Minimally worse,” 6 = “Much worse,” or 7 = “Very much worse.”) was recorded daily for 13 consecutive days starting the day after enrollment. Caregivers were asked to complete the GRCD by recording their responses directly into an Internet portal or on a paper-based version of the questionnaire (for those without Internet access) twice daily for 14 days—10 daytime symptoms were recorded in the evening (“since your child awoke this morning until you put your child to bed”) and 9 nighttime symptoms were recorded in the morning (“in the morning after your child has woken up for the day”). GRCD items are scored on 5- to 6-point ordinal rating scales assessing severity, with an additional option of “I don’t know” for items assessing overnight symptoms. The schedule of key events is included in the Additional file (Additional file 1: Table S2, online).

This study was reviewed and approved by the appropriate ethics committees. All caregivers provided written informed consent and parental permission.

### Analysis Methods

Item-level descriptive statistics and graphical techniques examined symptom prevalence and change over time and evaluated floor and ceiling effects. Principal components analysis and exploratory factor analysis (EFA) were conducted in an effort to understand the structure of the GRCD and determine an optimal scoring algorithm. Maximum likelihood estimation was used, and EFAs retaining varying numbers of factors were performed, with the decision as to the number of factors based on established criteria, the sizes and pattern of the factor loadings, and the interpretability of the factor(s) [15, 16].

#### Reliability

To document test-retest reliability, kappa coefficients and intraclass correlation coefficients (ICCs) were computed using the subset of patients assumed to be stable from day 13 (“test”) to day 14 (“retest”) because caregivers responded exactly the same on the PGIC on both days [17, 18]. It is recommended that kappa coefficients exceed 0.20 and ICCs be ≥0.70 for multi-item scales [19, 20]. Internal consistency reliability was evaluated by computing Cronbach’s coefficient alpha, where the approximate range of optimal alphas is between 0.70 and 0.90, indicating a set of strongly related but nonredundant items capable of supporting a unidimensional scoring structure [17, 21].

#### Validity

As evidence of construct validity, correlations were computed between GRCD scores and all clinician-reported (CGIS, clinical severity score) and caregiver-reported measures (PGIS, PGIC) [10, 22]. The goal was to demonstrate stronger relations among measures addressing similar constructs (convergent validity). For example, the GRCD was hypothesized to correlate more highly with the caregiver-reported PGIS than with the clinician-reported CGIS.

Known-groups analyses of variance (ANOVAs) and chi-square tests examined mean differences in GRCD scores between patients classified into groups on the basis of CGIS and PGIS scores, thereby providing evidence in support of the discriminating ability of the GRCD [23, 24]. Specifically, it was hypothesized that patients rated by clinicians as normal, borderline, or mildly ill would have lower GRCD scores compared with patients rated as moderately, markedly, severely, or among the most extremely ill patients. Similarly, patients rated by caregivers as mild or moderate on the PGIS were hypothesized to have lower GRCD scores compared with patients rated as severe or very severe on the PGIS.

To evaluate responsiveness, or the ability of the GRCD to detect change, effect sizes and standardized response means (SRMs) were calculated; effect sizes of approximately 0.20 are considered small, those of approximately 0.50 are moderate effects, and those greater than 0.80 are considered large [25].

## Results

The final analysis data set for the psychometric evaluation included 103 patient-caregiver pairs. Table 1 presents data describing the characteristics of participating patients and caregivers. All 103 completed the enrollment visit, and 102 (99.0%) caregivers completed the end-of-study telephone interview 15 to 18 days after enrollment; 20 (19.4%) caregivers completed all 14 days of the GRCD (overnight and daytime symptom diaries); 62 (60.2%) completed 10 or more days, and 89 (86.4%) completed the diaries for 7 days or more. On the first day of GRCD administration, 10% to 12% of overnight data were missing and 15% to 16% of daytime data were missing, depending on the item. At the seventh day of administration, missing data increased to 20.4% of overnight data and 21% to 22% of daytime data; at the last day of diary administration, 26% (overnight) to 27% (daytime) of responses were missing.

**Table 1:** Participant Characteristics

Characteristic	Baseline (*N* = 103)
Age (months), mean (SD)	7.41 (5.3)
0 to 6 months, n (%)	51 (49.5)
7 to 12 months, n (%)	35 (34.0)
13 to 24 months, n (%)	17 (16.5)
Sex—Female, n (%)	43 (41.7)
Race, n (%)	
White	76 (73.8)
Black or African heritage	11 (10.7)
Asian	6 (5.8)
American Indian, Alaskan Native, Native Hawaiian, or Pacific Islander	0 (0.0)
Other	2 (1.9)
Multiple races	8 (7.8)
Daycare—Yes, n (%)	43 (42.2)
Caregiver age (years), mean (SD), n	31.36 (5.5), 101
Caregiver sex—Female, n (%)	95 (93.1)
Caregiver race, n (%)	
White	80 (77.7)
Black or African heritage	11 (10.7)
Asian	6 (5.8)
American Indian, Alaskan Native, Native Hawaiian, or Pacific Islander	0 (0.0)
Other	2 (1.9)
Multiple races	4 (3.9)
Caregiver highest education level	101
Less than high school	1 (1.0)
High school or GED	13 (12.9)
Some college, technical training	19 (18.8)
College graduate	46 (45.5)
Advanced or professional degree	22 (21.8)

*SD* Standard deviation

### Item-Level Analyses

**Fig. 1:**
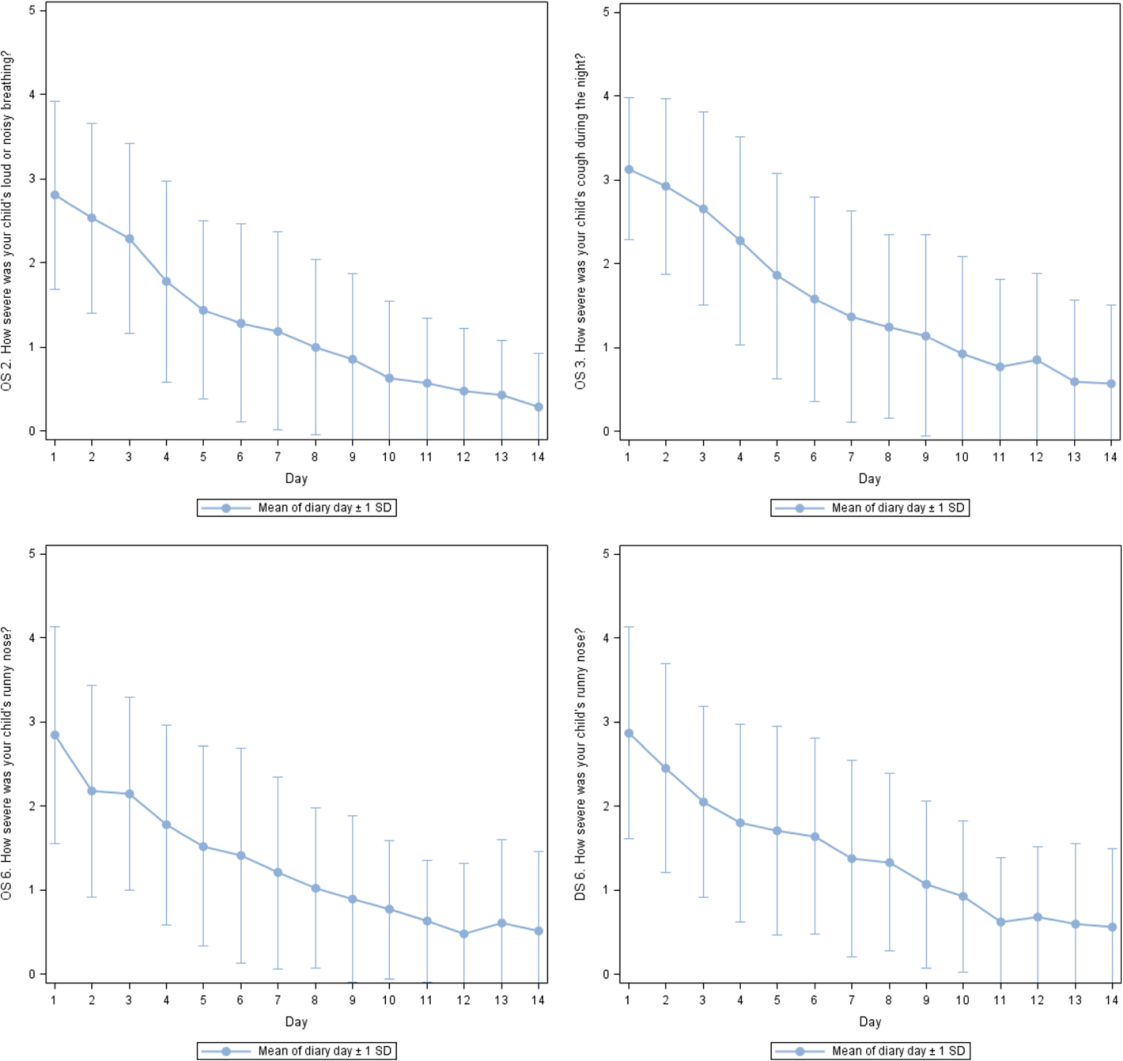
Item-level line plots displaying average item scores for four items over the course of the study, *SD* standard deviation

Item-level response distributions and descriptive statistics showed no evidence of response biases for any of the GRCD items. All GRCD items showed substantial improvement in overnight and daytime symptoms over the course of the 2-week data collection. Figure 1 displays the line plot of average item scores for four items: overnight loud or noisy breathing, overnight cough, overnight runny nose, and daytime runny nose.

Exploratory factor analysis was conducted using day 1 data. The initial principal components analysis of the day 1 data yielded seven eigenvalues greater than 1.0 (5.9, 2.4, 1.8, 1.6, 1.4, 1.3, and 1.1); based on the factor loadings, none of the solutions were interpretable. EFA was also conducted using day 14 data, but because the majority of caregivers reported no symptoms at day 14, the variability among patients at day 14 was extremely low; hence, factor loadings were not estimable due to a high degree of multicollinearity. Based on the qualitative research conducted during the development of the GRCD items, expert clinical input, and inter-item correlations, the GRCD subscale structure was expected to include a Respiratory Symptoms subscale with 8 items related to shallow breathing, noisy breathing, and cough; an RSV Symptoms subscale with 10 items related to shallow breathing, noisy breathing, cough, runny nose, and stuffy nose; a Behavior Impact subscale with 4 items on overnight sleep and daytime eating, activity level, and fussiness; and a Cough subscale with 4 items on daytime and overnight cough frequency and severity.

The inter-item correlation matrices (data not shown) showed patterns of weak, moderate, and strong correlations among sets of items that were used to define separate subscale scores. The Respiratory Symptoms subscale included 8 items related to shallow breathing, noisy breathing, and cough frequency and severity); the RSV Symptoms contained 10 items related to shallow breathing, noisy breathing, cough frequency and severity, runny nose, and stuffy nose; the Behavior Impact subscale included 4 items on overnight sleep and daytime eating, activity level, and fussiness; and the Cough subscale included 4 items on daytime and overnight cough frequency and severity.

Item-level test-retest reliabilities (data not shown) ranged in strength from poor (overnight fever kappa = − 0.00, daytime activity level kappa = − 0.02) to perfect agreement (daytime fever kappa = 1.00), with 17 of the 19 items achieving acceptable test-retest reliability.

Construct validity correlations between the GRCD items and the clinician-reported outcomes were generally weaker than expected (r = − 0.02 to 0.34), but correlations with the caregiver-reported PGIS were moderate to strong as hypothesized (r = 0.30 to 0.63) except for those associated with overnight fussiness (r = 0.29), overnight sleeping (r = 0.14), and overnight stuffy nose (r = 0.19). The correlations between item-level change from first day to last day and the PGIC at day 14 were generally moderate to strong, as hypothesized, except for a few very weak correlations with daytime fever (r = − 0.03), daytime shallow breathing (r = 0.01), overnight fever (r = 0.02), and overnight shallow breathing (r = 0.08). Although the exact patterns of weak, moderate, and strong correlations were not identical to what was hypothesized, relationships between GRCD items and the other measures were almost always in the anticipated direction and of the approximate size predicted. Known-groups analyses in support of item-level discriminating ability showed that means were typically higher for patients rated as more ill (84.2% of 57 ANOVAs), but few of these mean differences were statistically significant (14.0% of 57 ANOVAs).

With respect to responsiveness, item-level effect size estimates of change were large (data not shown), ranging from − 0.86 (overnight sleeping) to − 3.55 (daytime cough severity); SRMs were also large (data not shown), ranging from − 0.79 (overnight sleeping) to − 2.54 (daytime cough severity).

Based on the results of the item-level psychometric analyses, five items were deleted from the 19-item pilot version of the GRCD. The overnight fever item and daytime fever item were deleted due to poor reliability and construct validity correlations (r = 0.02 and r = − 0.03 with the PGIC, respectively), and relatively small responsiveness statistics (effect sizes = − 0.92 and − 0.96, respectively). Three overnight symptoms (overnight runny nose, overnight stuffy nose, and overnight fussiness) were eliminated due to discriminating ability results (all *P* > 0.05) and/or borderline validity correlations, while the matching daytime symptoms (daytime runny nose, daytime stuffy nose, and daytime fussiness) were retained, thereby reducing caregiver burden but not impairing the content validity of the GRCD.

### Subscale-Level Analyses

Guided by qualitative research and inter-item correlations, four subscale scores were created, in addition to a global GRCD score: an 8-item Respiratory Symptoms subscale (shallow breathing, noisy breathing, and cough), a 10-item RSV Symptoms subscale (shallow breathing, noisy breathing, cough, runny nose, and stuffy nose), a 4-item Behavior Impact subscale (overnight sleep and daytime eating, activity level, and fussiness), and a 4-item Cough subscale (daytime and overnight cough frequency and severity).

The subscale-level analyses evaluated different scoring rules for the GRCD subscales, one set based on the average of all daytime and overnight symptom ratings and another set based on the average of the maximum values of daytime and overnight symptom pair ratings. The best method for scoring the GRCD subscales involved the latter method, averaging the maximum values of symptom pair ratings (data not shown), based on two considerations.

First, the responsiveness statistics were typically somewhat better for the scores based on the averages of the maximum values (effect sizes = − 3.91 for the Global composite to − 4.40 for the Cough composite) compared with the scores based on the average of all ratings (effect sizes = − 3.64 for the Respiratory composite to − 4.22 for the Cough composite); responsiveness or sensitivity to change is an essential attribute of a COA. While the internal consistency reliabilities were larger for the scores based on the average of all daytime and overnight symptom ratings (range = 0.87 for the Respiratory composite to 0.94 for the Cough composite), the internal consistencies for the scores based on the averages of the maximum values were very satisfactory (range = 0.78 for the Respiratory composite to 0.94 for the Cough composite). There were no consistent or important differences between the two scoring methods in terms of test-retest reliability, validity correlations, or known-groups hypothesis tests.

Second, a scoring rule that involves selecting the maximum value of the daytime and overnight symptom pair is in keeping with the intent of the GRCD and the individual items—the items ask about the caregiver’s observation of the symptom at its worst during the day or night. In this way, the scoring of the GRCD subscales and global scores is aligned with the acute nature of the symptoms and the illness itself. The subscale-level results pertaining to the GRCD scores based on the average of the maximum values of symptom pair ratings are presented.

**Fig. 2:**
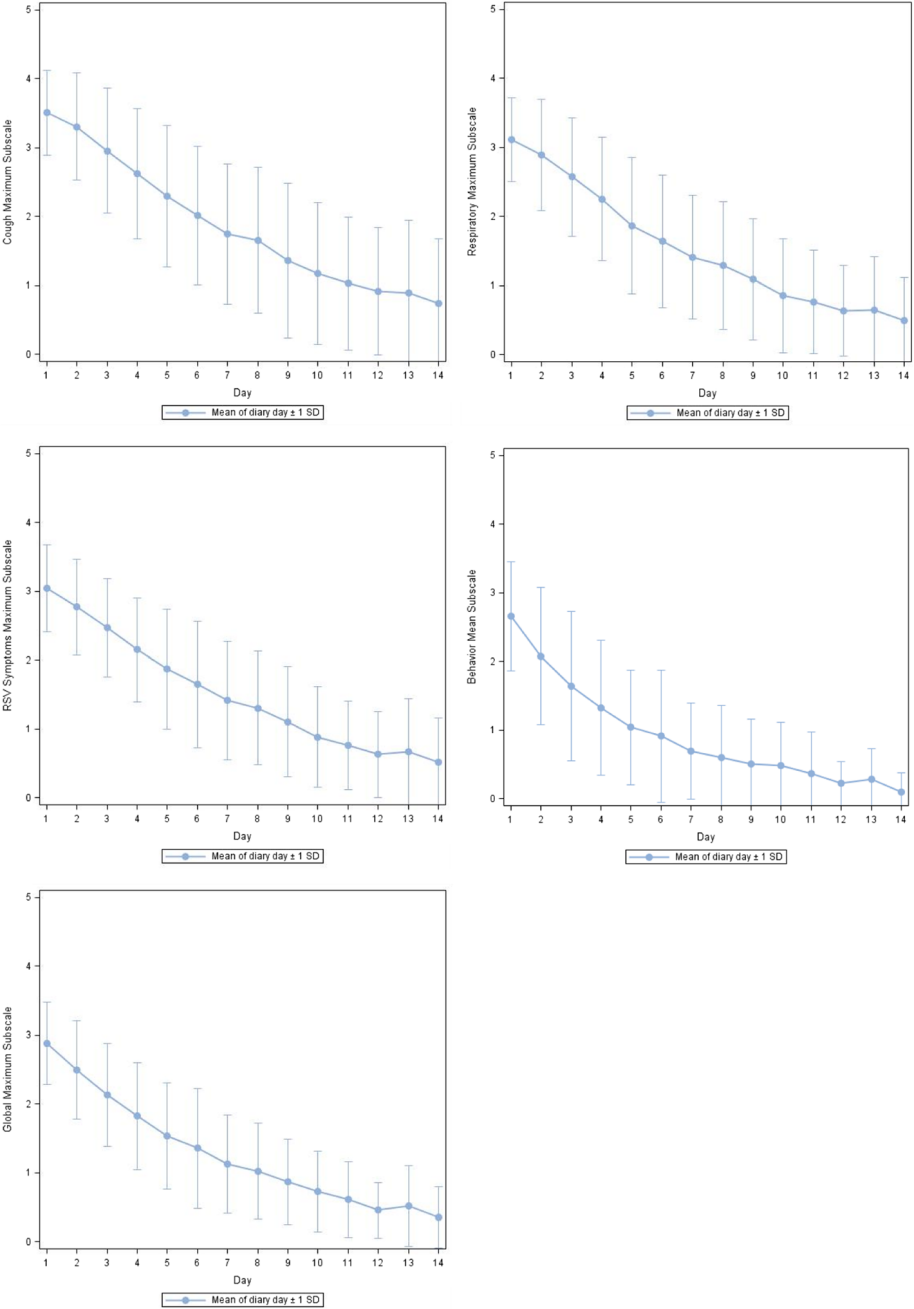
Subscale-level line plots displaying average GRCD subscale scores over the course of the study, GRCD = Gilead RSV Caregiver Diary; SD = standard deviation. Note: The Cough subscale included 4 items on daytime and overnight cough frequency and severity; the Respiratory subscale included 8 items on shallow breathing, noisy breathing, and cough frequency and severity; the 10-item RSV Symptoms subscale included shallow breathing, noisy breathing, cough frequency and severity, runny nose, and stuffy nose; and the 4-item Behavior Impacts subscale included overnight sleep and daytime eating, activity level, and fussiness

All subscale and global scores showed substantial symptom improvement over the course of the 2-week data collection. Figure 2 displays the line plot of GRCD subscale scores.

With respect to reliability, all test-retest ICCs (*n* = 39) for the GRCD subscales were above the recommended 0.7 value, except for the Behavior Impact subscale, which produced an ICC of 0.43 (Table 2). The internal consistency reliabilities (Cronbach’s alphas) ranged from 0.78 for the respiratory domain to 0.94 for the cough domain (Table 2).

**Table 2:** GRCD Scores—Reliability and Responsiveness

GRCD Domain	Day 13 to Day 14 ICC (95% CI)	Alpha Last Day	Effect Size^a^	Effect Size^b^	SRM^a^	SRM^b^
Cough	0.77 (0.61, 0.87)	0.94	−2.83	−4.40	−1.53	−2.72
Respiratory	0.81 (0.68, 0.90)	0.78	−2.73	−4.14	−1.69	−3.17
RSV symptoms	0.94 (0.89, 0.97)	0.84	−2.67	−4.16	−1.65	−3.16
Behavior impact	0.43 (0.13, 0.66)	0.84	−2.08	−2.70	−1.88	−2.82
Global	0.90 (0.82, 0.95)	0.84	−2.72	−3.91	−1.96	−3.49

*CI* Confidence interval, *ICC* Intraclass correlation coefficient, *SRM* Standardized response mean

^a^Computed using change from first day to seventh day

^b^Computed using change from first day to last day

Construct validity correlations followed expected patterns and supported the validity of the GRCD subscale and global scores (Table 3). The strongest correlations were between the GRCD scores and the other caregiver-reported measures, the PGIS and PGIC—all subscale and global scores except the Behavior Impact subscale achieved consistently strong correlations with the PGIS at day 1 and the PGIC at days 7 and 14. The correlations between GRCD scores and the clinician-reported CGIS and clinical severity scores were weak to moderate.

**Table 3:** Construct validity correlations (*n* = 49 to 78)

GRCD domain	CGIS	Temperature	Respiratory rate	Heart rate	Oxygen saturation	Clinical severity score	PGIS	PGIC Day 7^a^	PGIC Day 14^b^
Respiratory	0.08	0.06	0.03	0.07	−0.14	0.11	0.66*	0.68*	0.50*
RSV Symptoms	0.14	0.09	0.06	0.00	−0.14	0.10	0.70*	0.67*	0.63*
Behavior Impact	0.12	0.06	0.15	0.15	0.10	0.12	0.45*	0.43*	0.36
Cough	0.04	−0.03	−0.03	0.02	−0.02	−0.09	0.56*	0.66*	0.63*
Global	0.12	0.06	0.10	0.08	−0.03	0.12	0.68*	0.67*	0.62*

*CGIS* Clinical Global Impression of Severity, *PGIC* Parent Global Impression of Change, *PGIS* Parent Global Impression of Severity

*P < 0.01

^a^Correlations between PGIC day 7 and GRCD score change from first to seventh day of administration

^b^Correlations between PGIC day 14 and GRCD score change from first to last day of administration

Collectively, the known-groups analyses provided limited support for the discriminating ability of the GRCD (data not shown). All GRCD subscale and global scores differed significantly across subgroups of patients rated as less or more ill on the PGIS. For subgroups of patients based on the CGIS, mean differences were generally in the correct direction but not statistically significant.

Effect size estimates of responsiveness for the subscale and global score changes from the first day to the seventh day (range − 2.67 to − 2.83) and from the first day to the last day (range − 3.91 to − 4.4) were very large (Table 2).

## Discussion

A comprehensive set of RSV symptom items was developed in accordance with standards outlined in the FDA’s PRO guidance [10]. Development of the GRCD adheres closely to this guidance, by incorporating the specific input of caregivers of infants and very young children acutely infected with RSV and evaluating and documenting the psychometric properties of reliability, validity, and responsiveness of the GRCD item set in the same population. In addition, data from this prospective observational study were analyzed to inform item reduction in order to decrease respondent burden and develop a scoring algorithm for the GRCD.

Overall, the psychometric properties of the items, subscales, and total scores support the use and continuing refinement of the GRCD in clinical trials. Item-level test-retest reliabilities were acceptable for an ObsRO instrument, as were the subscale-level test-retest reliabilities (except for the Behavior Impact subscale). The internal consistency reliabilities of the GRCD were appropriate for its intended use, and inter-item correlations were generally as expected, providing evidence for construct validity. Correlational analyses supported the construct validity of the GRCD items, subscales, and total score and hypothesis tests in support of discriminating ability were in the anticipated direction and some were statistically significant, helping to verify the validity and usefulness of the GRCD. Responsiveness statistics were large, suggesting that the GRCD is capable of detecting change, a property that will be essential in future therapeutic trials.

### Limitations

One limitation of this study was the lack of measures available for a thorough evaluation of the construct validity of the GRCD, with no gold standard for the purpose of comparison. Not only are there no relevant disease-specific measures, but there are seemingly no parent- or caregiver-reported generic questionnaires of acute illness for infants and very young children with the short recall period necessary to show change over the relatively short time interval required for acute RSV infections. It is, however, possible that the administration of additional caregiver-reported measures would have presented an excessive burden to parents or caregivers of seriously ill children, and more questionnaires may have affected the response rate and increased missing data. As it was, the compliance rate was somewhat low and contributed to the decision to administer the GRCD once a day instead of twice a day in the future.

The weak to moderate correlations between GRCD scores and the clinician-reported CGIS and clinical severity scores are a potential limitation of the GRCD. However, while the literature shows that PRO measures often correlate more highly with other PROs than with clinician-reported measures [26–28] or with physiological measures [29], it is widely appreciated that the valuable data captured by patient-centered outcome measures is often independent of and complementary to clinical outcome assessments and enhances our understanding of the symptoms and impacts of diseases [30–33].

In addition, although the overall sample size in this study was sufficient for the estimation of important psychometric properties, the sample sizes for some of the subgroup analyses were small enough to adversely affect the power of the hypothesis tests. Future studies using the GRCD will undoubtedly use larger samples.

## Conclusions

The results of the present psychometric evaluation build on the qualitative research evidence for the GRCD and, while preliminary, support its reliability, validity, responsiveness, and usefulness for assessing the symptoms of RSV in an outpatient population [9]. The next step in documenting the validity evidence for the revised GRCD is to confirm the present results using a single daily administration, explore the potential for further item reduction, verify the scoring, and more thoroughly evaluate its construct validity in a therapeutic clinical trial. Responder definition thresholds will be estimated to characterize meaningful change and provide guidance on the interpretation of GRCD scores and change. The GRCD will be used and evaluated in future drug trials, with the expectation that it has the potential to collect important information from the parent or caregiver in a standardized manner capable of defining clinical improvement in RSV infection. This unique perspective can facilitate a more comprehensive evaluation of RSV disease symptoms and its treatment in clinical trials.

## Additional file

Additional file 1: **Table S1**. Inclusion/Exclusion Criteria for the GRCD Validation Study, **Table S2** Schedule of Key Events for the GRCD Validation Study. (DOCX 17 kb)

## References

[1] American Lung Association (2016) Learn About Respiratory Syncytial Virus (RSV). Available at: http://www.lung.org/lung-health-and-diseases/lung-disease-lookup/rsv/learn-about-respiratory.html. Accessed 16 Feb 2018.

[2] MedlinePlus (2016) Respiratory Syncytial Virus Infections. Available at: https://medlineplus.gov/respiratorysyncytialvirusinfections.html. Accessed 16 Feb 2018.

[3] Centers for Disease Control and Prevention (2016) Respiratory Syncytial Virus Infection (RSV). Available at: https://www.cdc.gov/rsv/about/symptoms.html. Accessed 16 Feb 2018.

[4] Santanello NC, Norquist JM, Nelsen LM, Williams VSL, Hill CD, Bisgaard H (2005). Validation of a pediatric caregiver diary to measure symptoms of postacute respiratory syncytial virus bronchiolitis. Pediatr Pulmonol.

[5] Jacobs B, Young NL, Dick PT (2000). Canadian acute respiratory illness and flu scale (CARIFS): development of a valid measure for childhood respiratory infections. J Clin Epidemiol.

[6] Barrett B, Locken K, Maberry R (2002). The Wisconsin upper respiratory symptom survey: A new research instrument for assessing the common cold. J. Fam Pract.

[7] Barrett B, Brown RL, Mundt MP (2009). Validation of a short form Wisconsin upper respiratory symptom survey (WURSS-21). Health Qual Life Outcomes.

[8] Obasi CN, Brown RL, Barrett BP (2014). Item reduction of the Wisconsin upper respiratory symptom survey (WURSS-21) leads to the WURSS-11. Qual Life Res.

[9] Lewis S, DeMuro C, Block S, Senders S, Wisman P, et al. (manuscript accepted for publication in the Journal of Patient-Reported Outcomes) Development of a novel observer-reported outcome measure for the assessment of respiratory syncytial virus (RSV) infection symptoms in pediatric clinical trials. 10.1186/s41687-018-0034-9 PMC5935018 29757334

[10] Food and Drug Administration (2009) Guidance for industry. Patient-reported outcome measures: use in medical product development to support labeling claims. Available at: http://www.fda.gov/downloads/Drugs/GuidanceComplianceRegulatoryInformation/Guidances/UCM193282.pdf. Accessed 16 Feb 2018. 10.1186/1477-7525-4-79 PMC1629006 17034633

[11] Midulla F, Scagnolari C, Bonci E (2010). Respiratory syncytial virus, human bocavirus and rhinovirus bronchiolitis in infants. Arch Dis Child.

[12] Guy W (1976) ECDEU assessment manual for psychopharmacology (United States Department of Health, education, and welfare publication no. 76-338). Rockville: National Institute of Mental Health.

[13] Lydick E, Yawn BP, Staquet MJ, Hays RD, Fayers PM (1998). Clinical interpretation of health-related quality of life data. Quality of life assessment in clinical trials—Methods and practice.

[14] Cappelleri JC, Zou KH, Bushmakin AG, Alvir JMJ, Alemayehu D, Symonds T (2014). Patient-reported outcomes—Measurement, implementation, and interpretation.

[15] Cattell RB (1966). The scree test for the number of factors. Multivariate Behav Res.

[16] Kaiser HF (1960). The application of electronic computers to factor analysis. Educ Psychol Meas.

[17] Streiner DL, Norman GR (1995). Health measurement scales: A practical guide to their development and use.

[18] Schuck P (2004). Assessing reproducibility for interval data in health-related quality of life questionnaires: Which coefficient should be used?. Qual Life Res.

[19] Landis JR, Koch GG (1977). The measurement of observer agreement for categorical data. Biometrics.

[20] Nunnally JC, Bernstein IH (1994). Psychometric theory.

[21] Cronbach L (1951). Coefficient alpha and the internal structure of tests. Psychometrika.

[22] Mokkink LB, Terwee CB, Patrick DL (2010). The COSMIN study reached international consensus on taxonomy, terminology, and definitions of measurement properties for health-related patient-reported outcomes. J Clin Epidemiol.

[23] Reeve BB, Wyrwich KW, Wu AW (2013). ISOQOL recommends minimum standards for patient-reported outcome measures used in patient-centered outcomes and comparative effectiveness research. Qual Life Res.

[24] Terwee CB, Bot SDM, de Boer MR (2007). Quality criteria were proposed for measurement properties of health status measures. J Clin Epidemiol.

[25] Cohen J (1988). Statistical power analysis for the behavioral sciences.

[26] Laugsand EA, Sprangers MA, Bjordal K, Skorpen F, Kaasa S, Klepstad P (2010). Health care providers underestimate symptom intensities of cancer patients: A multicenter European study. Health Qual Life Outcomes.

[27] Sprangers MA, Aaronson NK (1992). The role of health care providers and significant others in evaluating the quality of life of patients with chronic disease: A review. J Clin Epidemiol.

[28] Xiao C, Polomano R, Bruner DW (2013). Comparison between patient-reported and clinician-observed symptoms in oncology. Cancer Nurs.

[29] Rosenzweig JRC, Edwards L, Lincourt W, Dorinsky P, ZuWallack RL (2004). The relationship between health-related quality-related quality of life, lung function, and daily symptoms in patients with persistent asthma. Respir Med.

[30] Chen WC, McLeod LD, Nelson LM, Williams VSL, Fehnel SE (2014). Quantitative challenges facing patient-centered outcomes research. Expert Rev Pharmacoecon Outcomes Res.

[31] Doward L, Gnanasakthy A, Baker MG (2010). Patient reported outcomes: Looking beyond the label claim. Health Qual Life Outcomes.

[32] Calvert M, Kyte D, Mercieca-Bebber R, Slade A, Chan A-W, King MT (2018). Guidelines for the inclusion of patient-reported outcomes in clinical trial protocols — The SPIRIT-PRO extension. JAMA.

[33] European Medicines Agency Committee for Medicinal Products for Human Use (2016). Appendix 2 to the guideline on the evaluation of anticancer medicinal products in man: The use of patient-reported outcome (PRO) measures in oncology studies EMA/CHMP/292464/2014.

