# Supplementary material for: Psychometric evaluation of a caregiver diary for the assessment of symptoms of respiratory syncytial virus
Source: J Patient Rep Outcomes. 2018 Feb 21;2:10. doi: 10.1186/s41687-018-0036-7 (PMC5934931; doi:10.1186/s41687-018-0036-7)
Supplement: Supplementary file 1 — Table S1. Inclusion/Exclusion Criteria for the GRCD Validation Study, Table S2 Schedule of Key Events for the GRCD Validation Study. (DOCX 17 kb) [file 41687_2018_36_MOESM1_ESM.docx]

Supplemental Table 1. Inclusion/Exclusion Criteria for the GRCD Validation Study

Inclusion criteria:

- Parent/caregiver of a child aged less than 24 months at screening visit
- Parent/caregiver of a child of at least 37 weeks gestational age at birth
- Parent/caregiver of a child with a confirmed diagnosis of RSV infection (by rapid diagnostic) documented within 24 hours of enrollment visit
- Parent/caregiver able to have direct daily contact (≥ 14 hours of observation per day during the day and evening) with the RSV-infected child for 14 consecutive days after enrollment
- Parent/caregiver of a child who is being seen for the first physician-diagnosed acute respiratory tract illness with duration of symptoms ≤ 5 days (or worsening of chronic symptoms such as runny nose) at enrollment visit

Exclusion criteria:

- Parent/caregiver of a child who has a known chronic illness
- Parent/caregiver of a child who received any nonsteroidal immunosuppressive therapies within 12 months prior to screening or any systemic corticosteroids for > 7 consecutive days within 4 weeks prior to screening
- Parent/caregiver of a child who was hospitalized within the 28 days prior to the screening visit
- Parent/caregiver of a child who has previously documented presence of RSV in the last 14 days prior to enrollment

Supplemental Table 2. Schedule of Key Events for the GRCD Validation Study

| Procedures | Prestudy | Visit 1 (Day 1) | Days 2-14 | Days 15-18 | Additional Visit(s) |
| --- | --- | --- | --- | --- | --- |
| Confirm RSV via rapid antigen diagnostic | X |  |  |  |  |
| Obtain informed consent and parental permission | X |  |  |  |  |
| Obtain demographic information |  | X |  |  |  |
| Obtain vital signs and physical exam findings; complete CGIS |  | X |  |  | X |
| Review instructions for GRCD, PGIS, PGIC |  | X |  |  |  |
| Complete GRCD every morning and evening^a,b^ |  | X | X |  |  |
| Complete PGIS |  | X |  |  |  |
| Complete PGIC |  |  | X |  |  |
| End-of-study caregiver interview^c^ |  |  |  | X |  |
| Date of visit, reason for visit, and any treatments prescribed |  |  |  |  | X |

CGIS = Clinician Global Impression of Severity; GRCD = Gilead RSV Caregiver Diary; PGIC = Parent Global Impression of Change; PGIS = Parent Global Impression of Severity; RSV = respiratory syncytial virus.

^a^ The morning GRCD should be completed within 6 hours of the time the child woke up, and the evening GRCD should be completed within 6 hours of the time the child went to sleep.

^b^ Caregiver may begin with the evening entry on day 1 if child was enrolled outside the morning GRCD session window.

^c^ Completed by phone 15 to 18 days after enrollment visit.
